# The impact of electronic consultation on a Canadian tertiary care pediatric specialty referral system: A prospective single-center observational study

**DOI:** 10.1371/journal.pone.0190247

**Published:** 2018-01-10

**Authors:** Lillian Lai, Clare Liddy, Erin Keely, Amir Afkham, Julia Kurzawa, Nishard Abdeen, Tobey Audcent, Matthew Bromwich, Jason Brophy, Sasha Carsen, Annick Fournier, Leigh Fraser-Roberts, Hazen Gandy, Charles Hui, Donna Johnston, Kathryn Keely, Ken Kontio, Christine Lamontagne, Nathalie Major, Michael O’Connor, Dhenuka Radhakrishnan, Joe Reisman, Marjorie Robb, Lindy Samson, Erick Sell, William Splinter, Judy van Stralen, Sunita Venkateswaran, Kimmo Murto

**Affiliations:** 1 Department of Pediatrics, Division of Cardiology, Children’s Hospital of Eastern Ontario-Ottawa Children’s Treatment Center (CHEO-OCTC), University of Ottawa, Ottawa, Ontario, Canada; 2 Department of Family Medicine; C.T. Lamont Primary Healthcare Research Centre, Bruyere Research Institute, University of Ottawa, Ottawa, Ontario, Canada; 3 Department of Medicine, Division of Endocrinology and Metabolism, The Ottawa Hospital, University of Ottawa, Ottawa, Ontario, Canada; 4 The Champlain Local Health Integration Network, Ottawa, Ontario, Canada; 5 CHEO Research Institute, Ottawa, Ontario, Canada; 6 Department of Medical Imaging, CHEO-OCTC, University of Ottawa, Ottawa, Ontario, Canada; 7 Department of Pediatrics, CHEO-OCTC, University of Ottawa, Ottawa, Ontario, Canada; 8 Department of Surgery, Division of Otolaryngology, CHEO-OCTC, University of Ottawa, Ottawa, Ontario, Canada; 9 Department of Pediatrics, Division of Infectious Diseases, CHEO-OCTC, University of Ottawa, Ottawa, Ontario, Canada; 10 Department of Surgery, Division of Orthopedics, CHEO-OCTC, University of Ottawa, Ottawa, Ontario, Canada; 11 Department of Ophthalmology, CHEO-OCTC, University of Ottawa, Ottawa, Ontario, Canada; 12 Department of Psychiatry, CHEO-OCTC, University of Ottawa, Ottawa, Ontario, Canada; 13 Department of Pediatrics, Division of Hematology and Oncology, CHEO-OCTC, University of Ottawa, Ottawa, Ontario, Canada; 14 Department of Pediatrics, Division of Community Pediatrics, CHEO-OCTC, University of Ottawa, Ottawa, Ontario, Canada; 15 Department of Anesthesiology and Pain Medicine, CHEO-OCTC, University of Ottawa, Ottawa, Ontario, Canada; 16 Department of Pediatrics, Division of Pulmonology, CHEO-OCTC, University of Ottawa, Ottawa, Ontario, Canada; 17 Department of Pediatrics, Division of Neurology, CHEO-OCTC, University of Ottawa, Ottawa, Ontario, Canada; 18 Department of Anesthesiology and Pain Medicine, CHEO-OCTC, Roger Neilson’s House, Palliative Care Program, University of Ottawa, Ottawa, Ontario, Canada; 19 Department of Anesthesiology and Pain Medicine, CHEO-OCTC, CHEO Research Institute, University of Ottawa, Ottawa, Ontario, Canada; University of North Carolina at Chapel Hill School of Medicine, UNITED STATES

## Abstract

**Background:**

Champlain BASE^™^ (Building Access to Specialists through eConsultation) is a web-based asynchronous electronic communication service that allows primary-care- practitioners (PCPs) to submit “elective” clinical questions to a specialist. For adults, PCPs have reported improved access and timeliness to specialist advice, averted face-to-face specialist referrals in up to 40% of cases and high provider satisfaction.

**Objective:**

To determine whether the expansion of eConsult to a pediatric setting would result in similar measures of improved healthcare system process and high provider acceptance reported in adults.

**Design:**

Prospective observational cohort study.

**Setting:**

Single Canadian tertiary-care academic pediatric hospital (June 2014–16) servicing 1.2 million people.

**Participants:**

1. PCPs already using eConsult. 2.Volunteer pediatric specialists provided services in addition to their regular workload. 3.Pediatric patients (< 18 years-old) referred for none-acute care conditions.

**Main outcomes and measures:**

Specialty service utilization and access, impact on PCP course-of-action and referral-patterns and survey-based provider satisfaction data were collected.

**Results:**

1064 eConsult requests from 367 PCPs were answered by 23 pediatric specialists representing 14 specialty-services. The top three specialties represented were: General Pediatrics 393 cases (36.9%), Orthopedics 162 (15.2%) and Psychiatry 123 (11.6%). Median specialist response time was 0.9 days (range <1 hour-27 days), most consults (63.2%) required <10minutes to complete and 21/21(100%) specialist survey-respondents reported minimal workload burden. For 515/1064(48.4%) referrals, PCPs received advice for a new or additional course of action; 391/1064(36.7%) referrals resulted in an averted face-to-face specialist visit. In 9 specialties with complete data, the median wait-time was significantly less (p<0.001) for an eConsult (1 day, 95%CI:0.9–1.2) compared with a face-to-face referral (132 days; 95%CI:127–136). The majority (>93.3%) of PCPs rated eConsult as very good/excellent value for both patients and themselves. All specialist survey-respondents indicated eConsult should be a continued service.

**Conclusions and relevance:**

Similar to adults, eConsult improves PCP access and timeliness to elective pediatric specialist advice and influences their care decisions, while reporting high end-user satisfaction. Further study is warranted to assess impact on resource utilization and clinical outcomes.

## Introduction

The World Health Organization reports that excessive wait-times for specialist care can impact continuity of care, leading to inappropriate treatment and potential patient harm. Canada has the second longest wait time for adult specialists among 10 other commonwealth countries.[1] Dissatisfaction with the referral process in Canada is evident among both adult-based primary care practitioners (PCPs) and specialists.[2] Pediatric literature on Canadian wait times for specialist care is limited. While there is published data on surgical wait times,[3] no data exists on wait times for elective pediatric medical referrals. A 2015 internal review conducted at the tertiary care Children’s Hospital of Eastern Ontario-Ottawa Children’s Treatment Center (CHEO-OCTC), Ontario, Canada, however, revealed elective wait times of up to 16 months for some specialties (personal communication Dr. Ciaran Duffy, CHEO-OCTC April 2015), mirroring the excess wait times seen in adults. Dissatisfaction with the pediatric referral process is assumed to be similar to that found among adult-based providers, and supports the need for more timely access to specialist care for Canadian children.

Electronic consultation is a potential solution to address prolonged specialist wat-times that has been implemented by several countries to improve access to single and multiple adult-based specialty departments.[4–7] Research has shown that eConsult, defined as an asynchronous consultative provider-to-provider communication within a shared web-based platform or electronic health-record[7], has enabled more timely access to care in the adult population and high levels of satisfaction among providers and patients.[7–12] Although three integrated American health care systems figure prominently in the literature (San Francisco General Hospital, the Mayo Clinic and the Department of Veterans Affairs)[7], it is the Canadian Champlain BASE^™^ (Building Access to Specialists through eConsultation) eConsult service, here in referred to as “eConsult” and located in Ottawa, Ontario, who have reported the largest number of available specialty services and eConsult requests. This secure web-based platform enables PCPs to request advice from 84 different adult specialty groups. As of December 22, 2017, over 32,293 eConsults have been completed with a median response time of two days and averted a face-to-face (FTF) referral in up to 40% of cases.[13–15] With the aim to address the long wait times in our region and to contribute to the dearth of pediatric eConsult literature[16–19], in June 2014 the breadth of pediatric specialty services offered through BASE eConsult, which was initially limited to general pediatrics and hematology/oncology,[20] was expanded. The specific objectives of this study were to evaluate the impact of the expanded pediatric services available through eConsult on measures of PCP access to specialty services, elective hospital-referral patterns, provider satisfaction, and to estimate patient family cost-savings. Based on our adult experience with BASE eConsult, we hypothesized that PCP eConsult utilization would be high and impact patient care, 40% of eConsult referrals would avoid a FTF specialist visit, wait-times for specialist advice and elective hospital-referrals would decrease, provider satisfaction would be high, and cost-savings would be realized.

## Materials and methods

### Setting and participants

This study reports on the results of a 2-year pilot phase which began in June 2014 at a 150-bed tertiary care pediatric hospital (CHEO-OCTC) located within the Champlain local health integration network (LHIN) one of 14 LHINs within the Province of Ontario, Canada, that services approximately 1.2 million people, 269,056 (20.2%) of whom are 0–18 years old.[21] CHEO-OCTC provides complete pediatric specialty consultation to Ottawa and the surrounding region and locations outside of our LHIN (i.e. northern Ontario, Nunavut and Gatineau, Quebec).

Participants were PCPs (family doctors and nurse practitioners) already using eConsult representing approximately 75% all PCPs practicing within the Champlain LHIN. CHEO-OCTC clinician specialists and community pediatricians were recruited on a volunteer basis. The former did not receive additional income for participating due to restrictions of their salary arrangement. At least two specialists per service were targeted to ensure timely coverage of eConsult requests. The eConsult service was available for any pediatric patient from within the LHIN catchment area through their PCP for any medical condition not requiring acute care.

### The Champlain BASE eConsult service (eConsult)

The eConsult service is web-based and developed to allow PCPs to submit a patient-specific clinical question to a specialist, using a standardized electronic form. A detailed description of the eConsult process including issues related to privacy and security are provided in S1 Methods. Specialists are expected to respond within 1 week. As part of the case-closure process all PCPs are required to complete a close-out survey (S2 Methods) to assess impact, which includes written feedback to the specialist.

### Qualitative and quantitative outcomes: Provider survey responses, system utilization & projected cost-savings

The primary outcomes were the impact of eConsult on the PCP’s course of action and associated referral-pattern. Secondary outcomes reflected impact on specialty service wait-times, elective hospital specialty referral patterns, provider acceptance (e.g. satisfaction and perceived workload burden) and projected indirect family/caregiver cost-savings as a result of an averted FTF specialist clinic visit. All data was collected prospectively. For each case-file, several data points, including demographics, the clinical question asked and the types and content of answers provided were automatically captured and stored. Time-dependent data including log-in times, time spent on the consultation, time for reply and closure of the case were also recorded. Each specialist was required to complete a satisfaction survey (S3 Methods) after study closure.

Electronic health record (EPIC) and individual department databases were used to quantify the impact of eConsult on the number of hospital referrals received by specialists and wait times. The pre-program implementation period was defined as July 1, 2013 to May 31, 2014 (11months); the post-period was defined as June 1, 2015 to April 31, 2016 (11months) allowing for 1 year (June 2014-May 2015) of established use. Finally, family/caregiver indirect cost-savings projections were determined based on the number of FTF consults avoided and as a result avoided travel expenses and costs associated with lost wages/productivity. As such, the following assumptions were made: all parent/guardians worked; the time taken to complete a FTF clinic visit at CHEO-OCTC necessitated at least a half-full day off work (depending on urban or rural designation); and the family’s urban/rural designation was based on the PCP’s office location.

### Statistical analysis

The data were analyzed using SPSS 21 (SPSS, Inc. Chicago IL, USA). All data are summarized using descriptive statistics including proportions, means or medians where appropriate. The mean numbers of monthly referrals for the pre-and post-program implementation periods were compared using a two-sample t-test. Before performing the t-test, a test for equality of variances was used, verifying that the variances in the two samples were not significantly different (p = 0.63). Wait times were compared using the Mann-Whitney-U tests. A P value <0.05 was considered statistically significant.

### Ethics approval

Both the CHEO Research Institute’s Ethics Review Board (REB No. 13/225X and 17/206X) and the Ottawa Health Science Network Research Ethics Board (REB No. 2009848) approved this research. All participating providers signed a consent form when registering for the service. Separate specialist consent to assess their satisfaction with eConsult via a survey was obtained. Patients and/or parents/guardians did not sign consent. It was expected, as with any referral, that the provider has verbally informed the patient and/or parents/guardian they will be using the eConsult service to get specialist input into their case.

## Results

### Provider characteristics

Within a few months of initiation, 16 specialty services supported by 25 participating physician specialists were represented in the expanded pediatric BASE eConsult platform (Table 1). The participating PCPs consisted of family doctors (n = 318) and nurse practitioners (n = 49). Most PCPs were located in urban areas (87.5%) while the remainder practiced in a rural setting.

**Table 1:** Distribution of specialties and number of specialists providing eConsult service.

Participating Specialties	eConsults completed No. (%) (n = 1064)	No. of Specialists (n = 25)
**General Pediatrics**^a^	393 (36.9)	5^b^
**Orthopedics**	162 (15.2)	2
**Psychiatry**	123 (11.6)	2
**Hematology/Oncology**	85 (8.0)	1
**Neurology**	65 (6.1)	2
**Infectious Disease**	63 (5.9)	2
**Ophthalmology**	52 (4.9)	2
**Cardiology**	41 (3.9)	1
**Otolaryngology (ENT)**	26 (2.4)	1
**Refugee & Immigrant Health**	19 (1.8)	1^b^
**Pulmonology (Respirology)**	14 (1.3)	2
**Radiology**	13 (1.2)	1
**Chronic Pain**	7 (0.7)	1
**Anesthesiology**	1 (0.1)	1
**Palliative Care**	0 (0.0)	1
**Complex Care**	0 (0.0)	1
**Total**	1064 (100)	**25**

ENT, Ear Nose and Throat.

^a^In Canada, general pediatric services are considered a specialty consultation service, and therefore, were included as a specialty service; general pediatrics includes the attention deficit hyperactivity disorder service.

^b^One specialist provided service both as a”Refugee & Immigrant Health” and a “General Pediatrics” physician.

### Utilization of eConsult

A total of 1064 pediatric eConsult requests were directed to 14 of 16 specialty services (Table 1). The top three pediatric specialties consulted were General Pediatrics 393 cases (36.9%), Orthopedics 162 (15.2%) and Psychiatry 123 (11.6%). The majority of PCPs (55.9%) used the service 2 or more times with a median of 2 (range 1–31) eConsult requests per PCP. The median initial specialist response time to the PCP request was 0.9 days (range <1 hour to 27 days). The delays occurred early in our experience and were due to specialist absence without available back-up. Most specialists reported spending less than 10 minutes per eConsult (63.2% of cases specialists completed the case in <10min; 22.6% in 10-15min; 10.2% in 15-20min; 4.1% >20min). The specialist responded without need for further information in 983 (92.4%) cases. In the remaining 81 (7.6%) cases, either the specialist or PCP asked additional questions.

### Impact of eConsult on PCP course of action and referral patterns to the specialist

**Fig 1:**
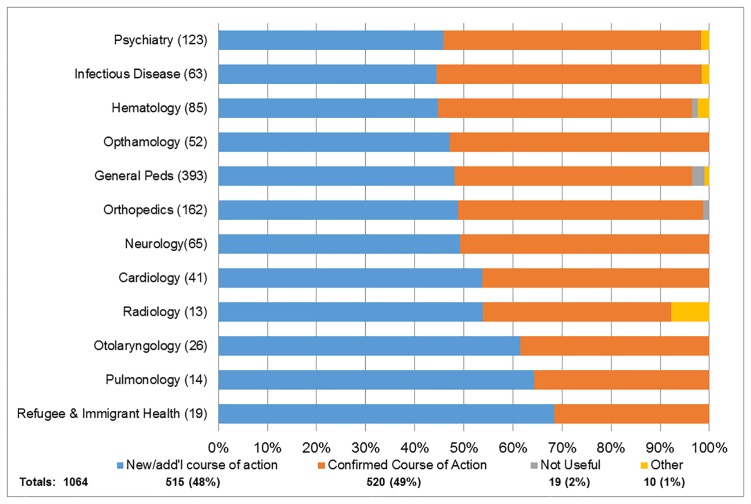
Impact on course of action by the primary care practitioner, by specialty service with > 10 cases. Impact of specialist response on PCP course of action: (Blue) Provided new information and/or additional course of action; (Red) Confirmed course of action; (Green) Considered not useful and (Purple) Other.

**Fig 2:**
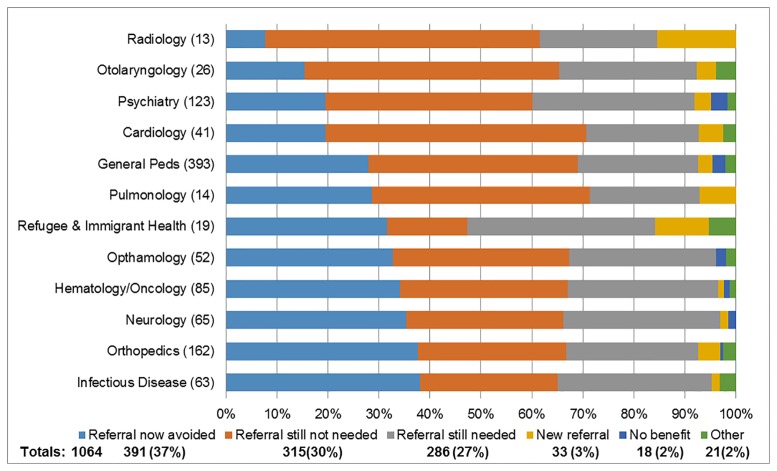
Impact on need for referral as indicated by primary care practitioner, by specialty service with > 10 cases. Impact of specialist response on PCP perceived need for patient referral: (Light Blue) Referral avoided; (Orange) Referral still not needed; (Gray) Referral still needed; (Yellow) New referral needed; (Dark Blue) No benefit and (Green) Other.

The impact of eConsult on the PCPs course of action is displayed in Fig 1. Overall, 515/1064 (48.4%) cases resulted in the PCP receiving good advice for a new or additional course of action, 520/1064 (48.9%) confirmed their original course of action and 19/1064 (1.8%) found eConsult “not useful”. The impact of eConsult on PCP referral activity is seen in Fig 2 (for associated comments, S1 Table). PCPs confirmed a FTF referral was still or still not required in 286/1064 (26.9%) and 315/1064 (29.6%) cases, respectively. Of note, 391/1064 (36.7%) of referrals were originally contemplated but were avoided as a result of using eConsult. Only 33/1064 (3.1%) of cases not thought to require a FTF referral by the PCP required FTF assessment by the specialist following eConsult.

### Impact of eConsult on specialty service wait-times

Nine specialties had complete wait-time data for comparative analysis. Sixteen observations were removed from analysis as they were recorded inaccurately in the hospital electronic health record (EPIC). The overall median wait-time of these nine specialty services for an eConsult (1 day; 95% CI 0.9–1.2) compared with a FTF referral (132 days; 95% CI 127–136) was significantly shorter (p<0.001) (Table 2).

**Table 2:** Comparison of specialist wait-time for eConsult versus elective referrals for nine specialties at CHEO-OCTC.

Specialty	eConsult			Face-to-Face			P Value
	Median Wait Time (days)	N	IQR (days)	Median Wait Time^a^ (days)	N	IQR (days)	
**Cardiology**	0.5	41	1.8	204	357	100	<0.001
**Otolaryngology**	1.8	26	4.6	313	550	294.3	<0.001
**Infectious Disease**	0.3	63	2.4	30	712	38.3	<0.001
**Neurology**	0.7	65	2.8	306	290	226	<0.001
**Ophthalmology**	3.6	52	5	482.5	60	589	<0.001
**Orthopedics**	0.9	162	3.6	188	291	45.5	<0.001
**General Pediatrics**	0.6	393	3.6	102	82	62.8	<0.001
**Psychiatry**	4.4	123	5.8	99	1163	140	<0.001
**Pulmonology**	8.8	14	13.4	126	892	72	<0.001

IQR,Interquartile range.

^a^16 observations were removed from analysis as they were recorded inaccurately in the hospital electronic health record (EPIC).

### Hospital elective-referral patterns

CHEO-OCTC began tracking referrals in July 2013 in a gradual rollout fashion. As a result, pre-and post-program implementation data of elective referral patterns was only available for 8 specialties (S1 Fig). During the study time period, CHEO-OCTC received 18,700 referrals, of which 3246 were triaged as “elective” referrals. There was minimal difference between the monthly rates of specialist visits (124 vs 123 referrals/month; P = 0.91) and total elective referrals (1520 vs 1448 cases) prior to and following study implementation.

### Satisfaction

There was a 1064/1064 (100%) survey response rate among PCPs. They felt that the overall value of the service was excellent or very good value for them (93.3%) and their patients (94.4%) (see examples of comments in S2 Table). The specialist satisfaction survey response rate was 21/25 (84%). The majority of specialist survey respondents (20/21) “strongly agreed” or “agreed” that the system was simple and easy to use. On workload, 5/21 (24%) felt there was no significant impact to clinical workload, while the majority 16/21 (76%) felt an acceptable increase in workload. No specialist felt it increased workload significantly. All specialist survey respondents indicated eConsult should continue as a hospital service provided to the community.

### Potential parent/caregiver cost-savings

The PCPs reported that eConsult averted 391 FTF visits. Estimated indirect cost savings for the patient families was approximately $135.16 CAN per visit for a total savings of $50, 895.00 based on 337 patients (54 patients outside of Champlain LHIN were excluded) (Table 3).

**Table 3:** Indirect parent/caregiver cost-savings within the Champlain region, by urban, rural and combined urban/rural designation (n = 337).

Metrics	Cost
Median Canadian hourly income^a^	$23.57 CAN/hr.
Work hours lost^b^	Urban: 4 hours; Rural: 8 hours
Ontario mileage reimbursement rate^c^	$0.55 CAN/KM
Median distance travelled by patients and families^d^	28.4 KM
Hospital Parking	$13.00 CAN
Avoided referrals	337
No. of patients in urban and rural designation	293/337 (86.9%) in urban 44/337 (13.06%) in rural
Urban family/caregiver cost savings: ($23.57 CAN/hr. X 4 hrs.) + (0.55 CAN/KM X 28.4 KM) + $13.00 CAN	$122.90 CAN
Rural family/caregiver cost savings: ($23.57 CAN/hr. X 8 hrs.) + (0.55 CAN/KM X 28.4KM) + $13.00 CAN	$217.18 CAN
Ratioed cost savings per family/caregiver: (0.869 X 122.9 CAN) + (0.1306 X 217.18 CAN)	$135.16 CAN
Total cost savings: 337 patients X $135.16 CAN	$50, 895.00 CAN

^a^Government of Canada. (2016)[31]

^b^ Ontario Ministry of Health and Long-Term Care[32]

^c^ Government of Canada. (2017)[33]

^d^ Based on location of PCP office to CHEO[32,34]

Note: Family/caregiver indirect cost-savings projections were based on 337 FTF consults (rural and urban) avoided; referrals requested within the Champlain region only, excludes all other areas.

## Discussion

This prospective cohort study details the impact of eConsult within an urban pediatric universal healthcare setting. Specifically, it demonstrated that the eConsult service significantly increased PCP access and timeliness to specialist advice for non-urgent referrals, with a high rate of satisfaction among health care providers. It deferred one-third of cases from requiring a FTF specialist visit, saving patient’s families an estimated $135 CAN per visit. System capacity for specialist visits was increased while reporting minimal associated workload burden.

Our results are comparable to the only other pediatric eConsult system, Electronic Children’s Hospital of the Pacific (ECHO-Pac), based out of the Tripler Army Medical Center in Hawaii.[18,19] Despite servicing different populations (civilian urban versus rural and military urban) we report similar high rates for surgical consultations requests and short wait times to access specialist advice. Our lower rates of eConsults per 1000 children per month, (0.17 versus 1.0) may be due to different directives provided to the users on cases and conditions eligible for eConsult services or the fact that their service was established for several years prior to their study.[16,18] Indeed, the number of pediatric eConsults were only half that observed over the same timeframe in the adult BASE eConsult service, suggesting that this service was not yet as established. In addition, we may not have accounted for the relative exclusion of enrolled community pediatricians who function as PCPs for some families and pediatric questions answered by adult-based specialists during this pilot period.

Our findings are comparable with adult studies in terms of high user satisfaction[13,22–24] with PCPs reporting improved and faster access, educational value, and avoidance of unnecessary patient travel. Compared to adult reports, we show similar rates of FTF referral avoidance, short access time to receive specialist advice[4,13] and specialist response times to answer an eConsult.[12,13,22] Our specialists’ high satisfaction mirrored the adult literature, where specialists report improved clarity of clinical questions, fewer inappropriate clinic visits, and improved efficiency by ordering more appropriate investigations making the first FTF visit more valuable.[7,22,25,26] However, some institutions find recruitment difficult due to concerns with increased workload and difficulty with technology. [7,22,25,26] While one of our specialists did cite technology as a barrier, they still felt eConsult should be an on-going service. Unique to the BASE eConsult system is the practice of providing the specialist with the written feedback from the PCP following the consultation. One specialist found this very satisfying compared to the lack of feedback from FTF consultations reported in the literature[9,27] and may explain the unanimous support for continued on-going provision of eConsult among specialists.

The eConsult team collectively answered 1064 eConsult referrals during this 2-year time period in addition to their regular clinic workload, thus, effectively increasing capacity in the system. Our finding that 12.5% of eConsults were from PCPs located in rural areas is consistent with the percentage of children living in rural areas in Ontario,[28] and may represent an important improvement in accessibility for this population. While previous studies have reported decreased hospital utilization following the implementation of electronic consultation,[29] our data did not demonstrate this in the 8 specialties for which complete pre-and post-implementation data was available. Essentially, hospital referral rates remained unchanged as indicated by the relatively flat trend line reported in S1 Fig. This may be due to the fact that the approximately 300 cases avoiding a clinic consult represented only 9% of all elective consults in the 8 specialties with complete data.

Our indirect cost-saving estimates suggest that patients and their families save approximately $135 CAN dollars and a half or full day off work or school for each avoided FTF consultation. This is a crude measurement because it does not account for eConsult-related direct costs consisting of those related to care delivery, consultation specific (e.g. added tests) and need for additional referrals.[30] To the 12.5% of eConsults from PCPs located in rural areas, eConsult may be an important health care service to reduce the time and money to support travel for FTF consultations.

We are the first to report on the use of a web-based electronic consultation portal to access multiple pediatric specialties in a mid-sized urban civilian setting within a universal healthcare access system. There are, however, several limitations to our study. First, despite being the largest reported so far in the pediatric literature our sample size is small, which may explain the lack of observed impact on resource utilization following implementation of the pediatric service. Second, this study is limited by the subjective PCP survey responses. We did not follow the patient to determine if they in fact avoided a FTF referral. A study is planned to track patients over the next 2 years using their universal health care number. Third, the impact of eConsult compared with a FTF specialist visit on costs related to resource utilization and clinical outcomes was not assessed; a cost-effectiveness analysis was beyond the scope of this study. Fourth, the high satisfaction seen in provider survey responses may be biased given that volunteer specialists likely already considered this service as useful and the community pediatricians were paid for their services. Furthermore, there was no direct assessment of patient satisfaction, which was only assessed via PCP opinion. Fifth, a true denominator of total referrals could not be calculated to put into context the impact of eConsult on referral activity. Despite these shortcomings, the short time for eConsultations (1 day) is nonetheless impressive and warrants further study.[35]

## Conclusion and future initiatives

In an urban civilian setting, pediatric eConsult improves access and timeliness to specialist advice, increases capacity in the system while providing high PCP and specialist satisfaction and saving the patient’s family time and resources. CHEO-OCTC has endorsed eConsult as a continuous service to be provided to our community. Furthermore, the Ontario Ministry of Health and Long-Term Care has recently decided to expand the delivery of this eConsult service to all 14 LHINs within the Province of Ontario. Future plans include assessment of the impact on patient outcomes related to efficacy, safety, stress and anxiety reduction, and management of vulnerable and isolated populations (e.g. Nunavut) including financial implications. Finally, we are exploring the nature of the questions posed by PCPs to define topics for their continuing medical education.

## Supporting information

S1 Methods The Champlain BASE^™^ eConsult service (eConsult). (DOCX)

S2 Methods Primary care practitioner close-out survey. (DOCX)

S3 Methods CHEO-OCTC specialist satisfaction survey. (DOCX)

S1 Table Examples of primary care practitioner comments associated with impact on referral, by category. Categories are “Need for new referral”, “No benefit” and ‘Other”.
(DOCX)

S2 Table Examples of theme-based comments by PCPs on close-out survey. Themes related to “Time saved”, “Money saved”, “Faster treatment” and “Reassurance”.
(DOCX)

S1 Fig Elective face-to-face referrals over pre-and post eConsult implementation period. Elective face-to-face referrals for July 2013-April 2016: (Blue) Number of referrals. (Red) Trend line.
(TIF)
