# Supplementary material for: The impact of electronic consultation on a Canadian tertiary care pediatric specialty referral system: A prospective single-center observational study
Source: PLoS One. 2018 Jan 10;13(1):e0190247. doi: 10.1371/journal.pone.0190247 (PMC5761872; doi:10.1371/journal.pone.0190247)
Supplement: S1 Methods — (DOCX) [file pone.0190247.s001.docx]

**S1 Methods. The Champlain BASE™ eConsult Service (eConsult)**

eConsult is a web-based service that was developed with the highest security features to allow PCPs to submit a patient-specific clinical question to a specialist, using a standardized electronic form[13,35] that allows for attachments (e.g. PDFs, bloodwork results and x-rays) to be included. PCPs sign a consent form upon signing into the system that acknowledges they are acting on behalf of their patient with the expectation that the patient will be informed of the results. A LHIN-based third party then assigns each e-consult to the appropriate specialty physician and sends an email alert to them with a link to a secure website portal. The specialist has the following four options: a) provide answers to questions and avoid a patient visit, b) request additional information before providing advice, c) recommend a formal referral with the option to recommend additional diagnostic tests or courses of treatment prior to the appointment or d) decline the request and/or recommend contacting a different specialty. E-mails are sent between the specialist and the PCP, indicating the status of the e-consult and allowing further communication. Specialists are expected to respond to an eConsult within one week. A permanent record of the e-consult can be downloaded for the patient’s file and accessed for review by the PCP and the specialist at any point thereafter. Specialists self-report their time spent responding to an e-consult and are paid a prorated hourly rate that was initially grant funded and is now Ministry of Ontario Health and Long-term Care funded. For the purposes of this study no physician working for the hospital was paid. This money was placed in a research fund. Four independent community pediatricians were directly remunerated while PCPs were not. The PCP is mandated to answer a survey to officially close the case-file (**S2 Methods**). The PCP written feedback is sent to the specialist for immediate review. All shared information is password protected and controlled to maximize patient confidentiality. The specifics of the secure Champlain LHIN-based platform, of which the main component is Microsoft SharePoint, can be found in detail, in previously published work.[29,35]
