# Supplementary figures and images for: The impact of electronic consultation on a Canadian tertiary care pediatric specialty referral system: A prospective single-center observational study
Source: PLoS One. 2018 Jan 10;13(1):e0190247. doi: 10.1371/journal.pone.0190247 (PMC5761872; doi:10.1371/journal.pone.0190247)

**S2 Methods. Primary care practitioner close-out survey**


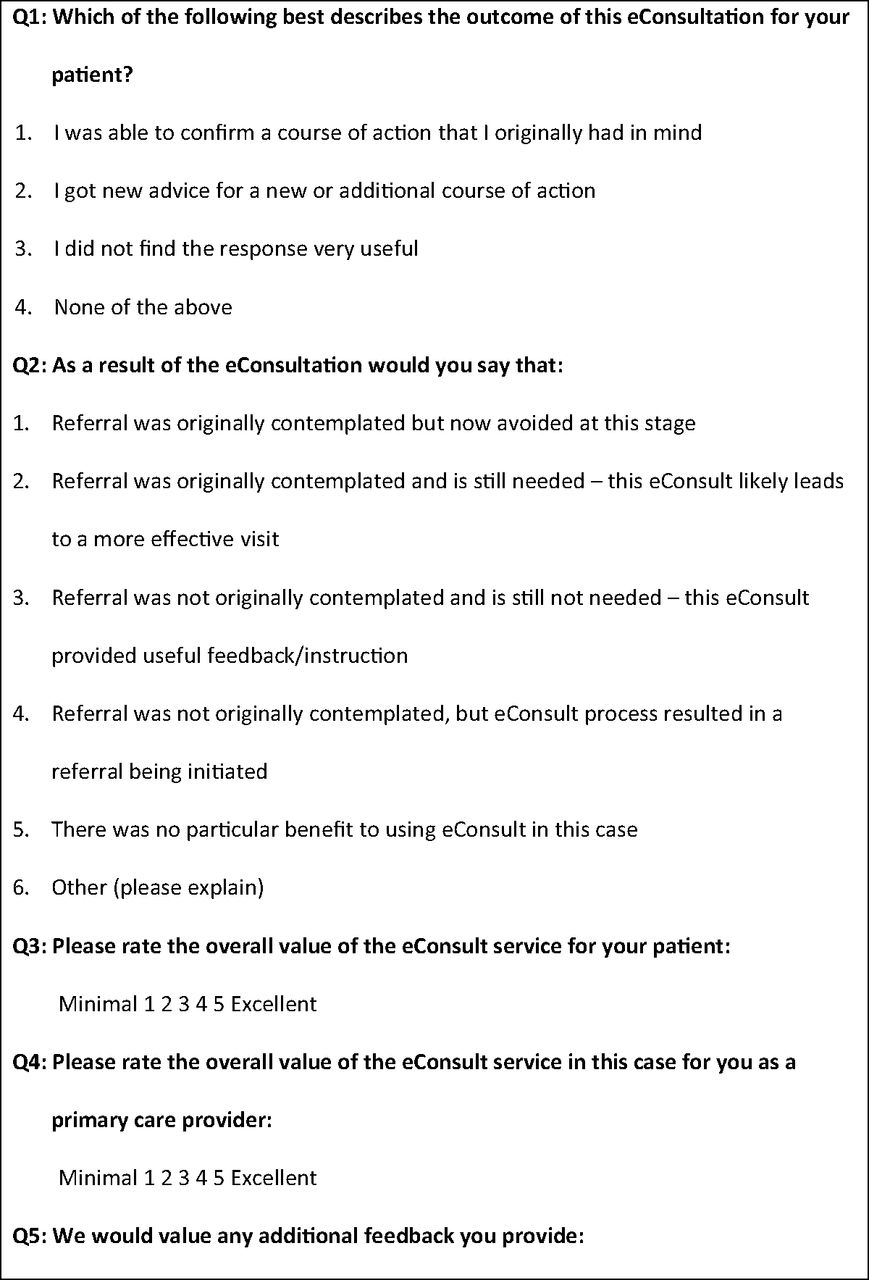

Supplement: S2 Methods — (DOCX) [file pone.0190247.s002.docx]

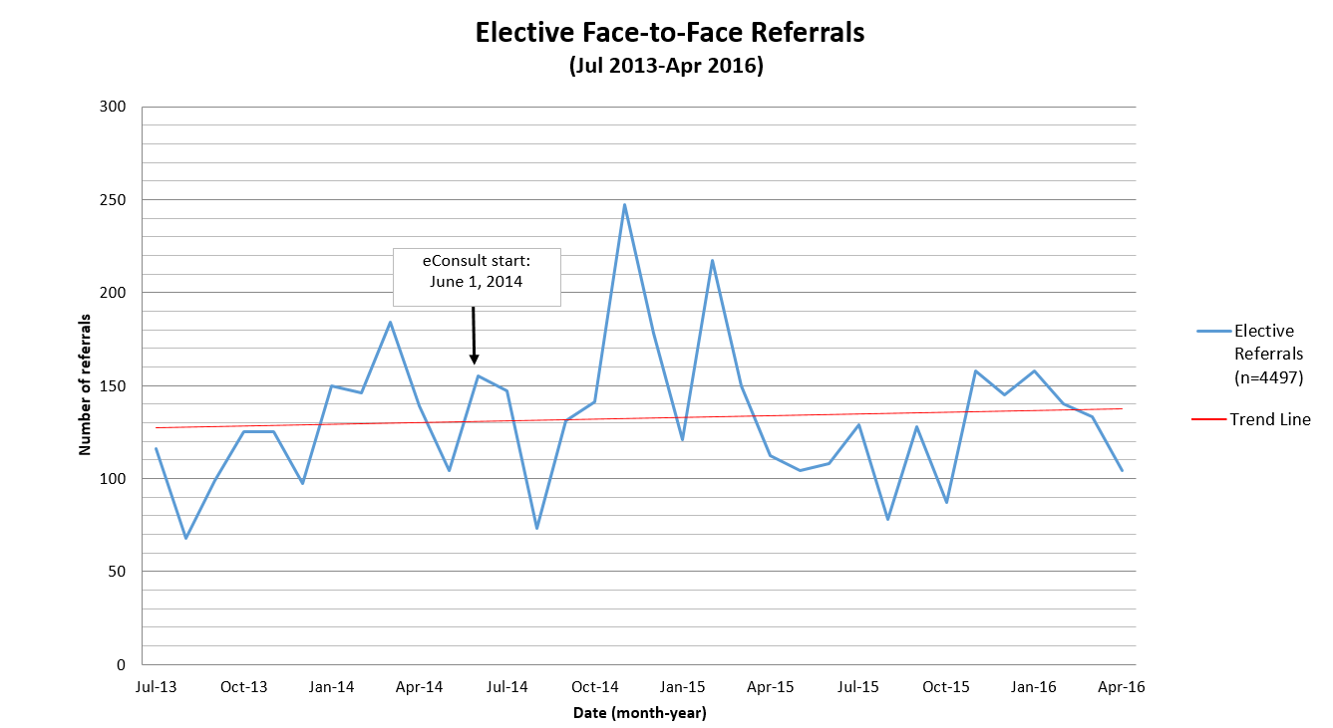

Supplement: S1 Fig — Elective face-to-face referrals for July 2013-April 2016: (Blue) Number of referrals. (Red) Trend line. (TIF) [file pone.0190247.s006.tif]
