# Supplementary material for: The impact of electronic consultation on a Canadian tertiary care pediatric specialty referral system: A prospective single-center observational study
Source: PLoS One. 2018 Jan 10;13(1):e0190247. doi: 10.1371/journal.pone.0190247 (PMC5761872; doi:10.1371/journal.pone.0190247)
Supplement: S3 Methods — (DOCX) [file pone.0190247.s003.docx]

**S3 Methods. CHEO-OCTC specialist satisfaction survey**

Specialist satisfaction survey

1. The eConsult system is simple and easy to use 1 2 3 4

(strongly agree-1, agree-2, disagree-3, strongly disagree-4)

a. if the system was not simple and easy, please explain why.

2. How has the introduction of eConsult affected your workload?

a. increased it significantly

b. increased it acceptably

c. no significant impact

d. decreased it

other comments:

3. Was the training for the use of eConsult appropriate? What could be done to improve that process?

4. What are the main problems with the system?

5. Was there strong technical support? If not please explain

6. How many times have you contacted technical support?

7. Do you think eConsult should continue to be implemented into the system as a consistent service to the LIHN?

a. Yes

b. No
