# Supplementary material for: The impact of electronic consultation on a Canadian tertiary care pediatric specialty referral system: A prospective single-center observational study
Source: PLoS One. 2018 Jan 10;13(1):e0190247. doi: 10.1371/journal.pone.0190247 (PMC5761872; doi:10.1371/journal.pone.0190247)
Supplement: S1 Table — Categories are “Need for new referral”, “No benefit” and ‘Other”. (DOCX) [file pone.0190247.s004.docx]

**S1 Table. Examples of primary care practitioner comments associated with impact on referral, by category**

| **PCP No. of Responses** | **Reasons for “Need for new referral” (n=31)** |
| --- | --- |
| 10 | unrecognized abnormal tests |
| 10 | unrecognized abnormalities in the history |
| 6 | unrecognized need for a clinical exam by the specialist |
| 5 | unrecognized need for specialist counseling |
|  | **Reasons for eConsult being of “No Benefit” (n=18)** |
| 4 | Too complex or too long of a wait for an urgent case |
| 2 | Technical issues |
| 1 | wrong service consulted |
| 1 | did not answer the question completely |
| 1 | eConsult told them to refer to an agency that has no ability to |
| 1  8 | reassurance received no change in management  No comments made |
|  | **“Other” comments(n=17)** |
| 8 | Good advice but only time will tell if a FTF consult is needed |
| 5 | positive comments suggesting that eConsult enabled patients to start medication sooner and that imaging was often avoided |
| 3 | Technical issues |
| 1 | Too long of a response (5 days) |

Note: see Figure 2 for associated quantitative data; PCP, primary care practitioner.
