# Supplementary material for: The impact of electronic consultation on a Canadian tertiary care pediatric specialty referral system: A prospective single-center observational study
Source: PLoS One. 2018 Jan 10;13(1):e0190247. doi: 10.1371/journal.pone.0190247 (PMC5761872; doi:10.1371/journal.pone.0190247)
Supplement: S2 Table — Themes related to “Time saved”, “Money saved”, “Faster treatment” and “Reassurance”. (DOCX) [file pone.0190247.s005.docx]

S2 Table. Examples of theme-based comments by PCPs on close-out survey

| **Underlying Theme** | **PCP Comment** |
| --- | --- |
| Time saved  Money saved | “…saved the patient having to travel to see a specialist which is really helpful given that she has 2 young kids and doesn't drive” |
| Time saved  Faster treatment | “The patient is on a 10 month wait list for a face-to-face visit so I will now be able to start some treatment at the suggestion of the psychiatrist. The patient and the family are very grateful. Thank you.” |
| Time saved  PCP and parent reassurance | “A referral was recommended and the detailed information about which clinic to refer to is invaluable. I had no idea that there was a clinic like this. This will save the parent/patient an appointment and get them directly to who they need to see. Very reassuring for both me and the parents.” |
| PCP reassurance | “Allowed me to feel confident in managing a clinical situation a little outside my scope... over a long weekend at that!” |
| Time saved  Money saved  PCP reassurance | “Great service. Especially helpful with things that I think are not a problem but I'm not absolutely sure.  Saves the patient and the system wasted time and money.” |
| Time saved | “Very helpful, especially because wait list for psych would be so long. Thanks!” |

PCP, primary care practitioner.
